# An Improved STARFM with Help of an Unmixing-Based Method to Generate High Spatial and Temporal Resolution Remote Sensing Data in Complex Heterogeneous Regions

**DOI:** 10.3390/s16020207

**Published:** 2016-02-05

**Authors:** Dengfeng Xie, Jinshui Zhang, Xiufang Zhu, Yaozhong Pan, Hongli Liu, Zhoumiqi Yuan, Ya Yun

**Affiliations:** 1 State Key Laboratory of Earth Surface Processes and Resource Ecology, Beijing Normal University, Beijing 100875, China; xiedfeng@mail.bnu.edu.cn (D.X.); zhuxiufang@bnu.edu.cn (X.Z.); pyz@bnu.edu.cn (Y.P.); 201521190003@mail.bnu.edu.cn (H.L.); miki@mail.bnu.edu.cn (Z.Y.); 201421190041@mail.bnu.edu.cn (Y.Y.); 2 College of Resources Science and Technology, Beijing Normal University, Beijing 100875, China

**Keywords:** unmixing-based method, spatial and temporal adaptive reflectance fusion model (STARFM), heterogeneity, data fusion, Landsat 8, MODIS

## Abstract

Remote sensing technology plays an important role in monitoring rapid changes of the Earth's surface. However, sensors that can simultaneously provide satellite images with both high temporal and spatial resolution haven’t been designed yet. This paper proposes an improved spatial and temporal adaptive reflectance fusion model (STARFM) with the help of an Unmixing-based method (USTARFM) to generate the high spatial and temporal data needed for the study of heterogeneous areas. The results showed that the USTARFM had higher accuracy than STARFM methods in two aspects of analysis: individual bands and of heterogeneity analysis. Taking the predicted NIR band as an example, the correlation coefficients (*r*) for the USTARFM, STARFM and unmixing methods were 0.96, 0.95, 0.90, respectively (*p*-value < 0.001); Root Mean Square Error (RMSE) values were 0.0245, 0.0300, 0.0401, respectively; and ERGAS values were 0.5416, 0.6507, 0.8737, respectively. The USTARM showed consistently higher performance than STARM when the degree of heterogeneity ranged from 2 to 10, highlighting that the use of this method provides the capacity to solve the data fusion problems faced when using STARFM. Additionally, the USTARFM method could help researchers achieve better performance than STARFM at a smaller window size from its heterogeneous land surface quantitative representation.

## 1. Introduction

High spatial and temporal resolution remote sensing technology plays an important role in land-cover detection, crop growth monitoring and phenological parameter inversion [1]. Unfortunately, it is impossible to obtain high temporal resolution and high spatial resolution images simultaneously from one sensor mounted on a satellite [2,3]. For example, Landsat series multi-spectral images at 30-m resolution have wide applications in extracting vegetation indices, monitoring land cover dynamic changes and ecological system variation studies. This system is widely used because of its finer spatial resolution, rich archive, and free availability [4,5,6]. However, the 16-day revisit cycle and the influence of bad weather, such as rain and clouds, make it difficult to acquire the continuous and cloudless remote sensing images that may be required to monitor certain Earth surface changes [7,8]. The Moderate Resolution Imaging Spectroradiometer (MODIS) onboard the Terra/Aqua satellites can provide remote sensing images at 1-day temporal, 250–1000 m spatial resolution, which is a potential alternative used in monitoring the Earth surface land coverage at large scales [9,10,11]. However, the low spatial resolution of MODIS data confines its application in fragmented and extremely heterogeneous landscapes [12]. If high spatial resolution and temporal resolution characteristics could be achieved at the same time, the advantages of Landsat and MODIS would be integrated and significantly improve the applicability of remote sensing technology to monitor land surface changes [13,14].

In recent years, researchers have developed a series of data fusion models to generate high spatial and temporal resolution remote sensing data. The unmixing-based method is one of these methods, which utilizes the Linear Spectral Mixture (LSM) model to extract endmembers reflectance at sub-pixel scale [15]. This method has previously been applied to fuse MODIS and Landsat images to produce high temporal and spatial resolution data [16,17,18,19,20,21,22]. Liu *et al.* [23] utilized the model to unmix Thermal Infrared Radiance for subpixel land surface temperature retrieval, and Wu *et al*. [24] proposed the MSTDFA model based on the LSM theory to generate daily synthetic Landsat imagery by combining Landsat and MODIS data. The Spatial and Temporal Adaptive Reflectance Fusion Model (STARFM) proposed by Gao *et al.* [25] is another effective fusion method to predict the phenology reflectance changes. Xu *et al.* [26] proposed a regularized spatial unmixing (RSpatialU)-based method, which introduced the prior class spectra estimated by STARFM to reduce the unmixing method error among the mixed pixels. The combination of the unmixed-based model and STARFM can utilize the advantages of the two modes to produce more accurate fusion data. Inspired by STARFM, Gevaert *et al.* [22] proposed a method entitled Spatial and Temporal Reflectance Unmixing Model (STRUM) which incorporates STARFM and unmixing-based fusion methods by combining the temporal change information obtained from the residue of two date MODIS images to improve the accuracy of unmixing-based method based on Bayesian theory. The fusion data generated by these methods are applicable to research in crop monitoring [20], environmental process detection [27], and mapping of forest disturbance [28], *etc.*

STARFM originated from the idea of capturing quantitative changes in radiometry caused by phenology through fusion of Landsat and MODIS data and has been widely applied for land surface detection. The method is applied to predict Landsat-like daily surface reflectance with one or several pairs of Landsat and MODIS images acquired on the same day and MODIS observations from the predicted date by weighing similar pixels for the central pixel [25]. The STARFM considers both the discrepancy of space and spectrum reflectance difference from multi-temporal MODIS images and has been widely introduced to produce high spatial-temporal data for detecting gradual changes over large areas [3,25,28,29]. However, several limitations for STARFM should be noted: it is incapable of estimating the transient or mutated surface changed information which is not captured by the base Landsat images [25,28,30]. STARFM also depends on temporal information from pure homogenous patches of land cover at the MODIS pixel scale and the predicted results can be misleading when used in heterogeneous landscapes, including cases of small-scale agriculture [25,28]. Many improvements of data spatial-temporal fusion models were conducted for STARFM. The Spatial Temporal Adaptive Algorithm for Mapping Reflectance Change (STAARCH), developed by Hilker *et al.* [28], was designed to detect the date on which land-cover change occurs and to record this information in a Landsat image to improve the final predicted result of the original STARFM approach [26,30]. For the latter limitation of STARFM, it is more difficult to find the pure MODIS pixels for central pixels in the fragmented area due to low resolution of MODIS when there are multiple land covers within one pixel. Zhu *et al.* [30] also attempted to develop an Enhanced STARFM method (ESTARFM) for heterogeneous landscapes regions, which introduces a conversion coefficient to succeed in enhancing the performance of prediction for heterogeneous landscapes. However, the precondition of at least two pairs of fine and coarse spatial resolution images on the same day for this method increases the difficulty of data acquisition and limits its applicability [30].

To resolve the difficulties that STARFM presents from the mixed pixel of MODIS in heterogeneous regions, we developed an improved STARFM named Unmixing-based STARFM (USTARFM) with the help of an Unmixing-based algorithm. The main objectives of this study include: (1) improve the performance of STARFM in heterogeneous areas, through unmixing the coarse pixel to gain specific land cover reflectance as the basis for further fusion rather than directed resampled data; (2) compare the fusion results of USTARFM and STARFM at different window sizes, and (3) assess the influence of landscape heterogeneity on the fusion performance of USTARFM. The USTARFM approach was applied to generate Landsat-like resolution and MODIS-like frequency images, and was tested using the Landsat 8/7 and MODIS reflectance as the reference data in two study areas.

## 2. Description of USTARFM

**Figure 1:**
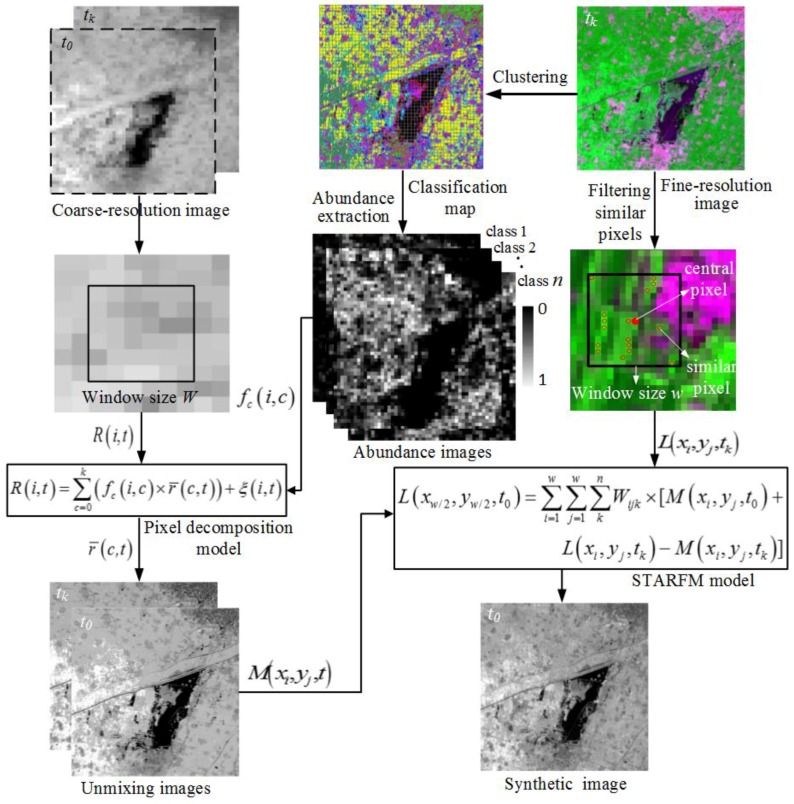
Flowchart of the USTARFM algorithm.

The coarse image on date *t*_0_ (predicted date) and a pair of fine-resolution and coarse-resolution remote sensing images on the same date *t_k_* (basis date) are needed for the USTARFM algorithm. Incorporating the clusters obtained from the fine resolution image on the *t_k_* as input component definition, the coarse resolution images (*t*_0_ and *t_k_*) were unmixed to get the land cover cluster reflectance. Unmixing data was used in place of the directly resampled data for the STARFM. The subsequent steps, like the STARFM, were run to predict the Landsat-like image. A sketch map of the USTARFM algorithm is shown in Figure 1, and the detailed implementation steps are listed as follows.

### 2.1. Land Cover Cluster and Abundance Extraction

The class types and related abundance within the MODIS mixed pixel are the two fundamental parameters for unmixing mixed pixels. The ISODATA algorithm has some further refinements by splitting and merging of clusters. Clusters are merged if either the number of members (pixel) in a cluster is less than a certain threshold or if the centers of two clusters are closer than a certain threshold. The ISODATA method has been used in many previous studies [1,18,19,20,21]. In our study, land cover was clustered using this method. The abundance of each class within MODIS pixel was calculated by summing the number of pixels occupied by each class and then we compared that to the total pixel number of coarse pixels [31,32]. The calculated abundance of each class and the class types were assumed constant during the prediction period [18,19,31], which is the precondition to downscaling the low resolution images at different dates (*i.e.*, *t_k_* and *t*_0_).

### 2.2. Unmixing Data

Mixed pixel decomposition based on the linear spectral mixture model is a popular method for estimating land cover fraction [15]. The surface reflectance of mixed pixels can be calculated as the sum of mean reflectance values of the different land-cover classes within the pixel, weighted by the corresponding abundance [33]. The abundance of classes and surface reflectance within coarse pixels (usually in window size) as input parameters can be solved with the ordinary least squares technique, producing the mean surface reflectance value ($\bar{r}$) for each land cover component as shown in Equation (1). Then mean reflectance values are assigned to each fine-resolution pixel of the classification map according to the class labels.

The spectral unmixing model is sensitive to the co-linearity caused by high correlations between endmembers, leading to inversion of ill-posed matrices [34]. This problem could be decreased by increasing the number of equations through enlarging the search window to adopt more classes, *i.e.*, increasing *n* in Equation (1) [22]. Furthermore, due to the existence of spatial heterogeneity, the solution of Equation (1) should also be conducted in a rectangular window, which can preserve spatial heterogeneity to the extent [31]. The oversize window scale containing too many pixels to make the model weakens spatial variation and leads to degradation in the accuracy of mixed pixel decomposition [1,31]. Hence, an appropriate window size is crucial to the decision of the coarse pixels decomposition. A set of different window sizes were tested to determine the optimal window size, and the optimal window size was measured with three metrics: the correlation coefficient (γ), Root Mean Square Error (RMSE), and the Erreur Relative Globale Adimensionalle de Synthèse (ERGAS) between the synthetic image and the reference fine-resolution image [1,31,35]:

(1) $$\begin{array}{c} \left[\begin{array}{c} R(1,t)\\ \ldots \\ R(i,t)\\ \ldots \\ R(n,t) \end{array}\right]=\left[\begin{array}{ccccc} f_{c}(1,1) & \ldots & f_{c}(1,c) & \ldots & f_{c}(1,k)\\ \ldots & . & . & . & \ldots \\ f_{c}(i,1) & \ldots & f_{c}(i,c) & \ldots & f_{c}(i,k)\\ .. & . & . & . & \ldots \\ f_{c}(n,1) & \ldots & f_{c}(n,c) & \ldots & f_{c}(n,k) \end{array}\right]\left[\begin{array}{c} \bar{r}(1,t)\\ \ldots \\ \bar{r}(c,t)\\ \ldots \\ \bar{r}(k,t) \end{array}\right]+\left[\begin{array}{c} \xi (1,t)\\ \ldots \\ \xi (c,t)\\ \ldots \\ \xi (k,t) \end{array}\right]\\ \text{constraints}:\sum_{c=0}^{k}f_{c}(i,c)=1;\;0<\bar{r}(c,t)<1 \end{array}$$

where R(i,t) is the reflectance of coarse-pixel *i* at time *t*, *f_c_*(*i,c*) is the abundance of class *c* in the *i_th_* coarse pixel, $\bar{r}\left(c,t\right)$ is the mean reflectance of class *c* at time *t*, ξ(i,t) is the residual error term, and *k* is the number of classes, *n* is the total coarse pixels number within the predefined window.

Apparently, the class number and the window size in Equation (1) are key parameters for the unmixing-based method. Therefore, by varying the class number (*k*) and the sliding window size (*W*), a series of unmixing data can be obtained and assessed by the metrics of quantative assessment indices. The optimal combination (*k,W*) of class number and window size was finally determined to unmix the MODIS scale resolution images [1].

### 2.3. Fused Image Generation

The two unmixed images from MODIS on date *t_k_* and *t*_0_, and one fine-resolution image on date *t_k_* are applied to predict the fine-resolution image on date *t*_0._ The critical step to implement the USTARFM algorithm, as well as the STARFM, is weighting the spatial information from neighboring pixels which are used to estimate reflectance of the central pixel and allow the weight function to be flexibly adjusted according to land surface complexity and heterogeneity [25]. To ensure that suitable information is acquired from neighboring pixels, similar spectral pixels from fine-resolution images within the moving window are involved to compute the reflectance of the central pixel. Then these similar pixels are weighed and used to calculate the reflectance of the central pixel as shown in Equation (3).

(2) $$W_{ijk}=\left(\frac{1}{S_{ijk}\times T_{ijk}\times D_{ijk}}\right)/\sum_{i=1}^{w}\sum_{j=1}^{w}\sum_{k=1}^{n}\left(\frac{1}{S_{ijk}\times T_{ijk}\times D_{ijk}}\right)$$

(3) $$L\left(x_{w/2},y_{w/2},t_{0}\right)=\sum_{i=1}^{w}\sum_{j=1}^{w}\sum_{k=1}^{n}W_{ijk}\times \left(M\left(x_{i},y_{j},t_{0}\right)+L\left(x_{i},y_{j},t_{k}\right)-M\left(x_{i},y_{j},t_{k}\right)\right)$$

where *L*(*x_w_*_/2_,*y_w_*_/2_,*t*_0_) represents the central pixel estimated reflectance of the moving window *w* at Landsat-like scale, *L*(*x_i_*,*y_j_*,*t_k_*) and *M*(*x_i_*,*y_j_*,*t_k_*) are the given location ( *x_i_*,*y_j_* ) pixel surface reflectance of the fine resolution image and unmixing data at the base date *t_k_* and *M*(*x_i_*,*y_j_*,*t*_0_) is the value of the unmixing data of MODIS on the prediction date *t*_0_. The *W_ijk_* refers to the weight of similar pixel (*x_i_*,*y_j_*,*t_k_*) , which is determined by three indices: spectral difference (*S_ijk_*), temporal difference (*T_ijk_*) and spatial distance between the central pixel and the similar pixel (*D_ijk_*).

A smaller value of *S_ijk_* implies that the fine-resolution pixel has spectral features that are similar to the coarse pixel, and thus, pixel reflectance should be assigned a higher weight in Equation (2). A smaller *T_ijk_* means there is less change in the surface reflectance between *t_k_* and *t*_0_; thus, the pixel should be assigned with a higher weight. A smaller *D_ijk_* means there is a closer distance to the central pixel, and therefore a higher weight for the pixel should be assigned.

For a more detailed description of the above algorithm readers may refer to [25].

## 3. Algorithm Test

### 3.1. Test Data and Preprocessing

**Figure 2:**
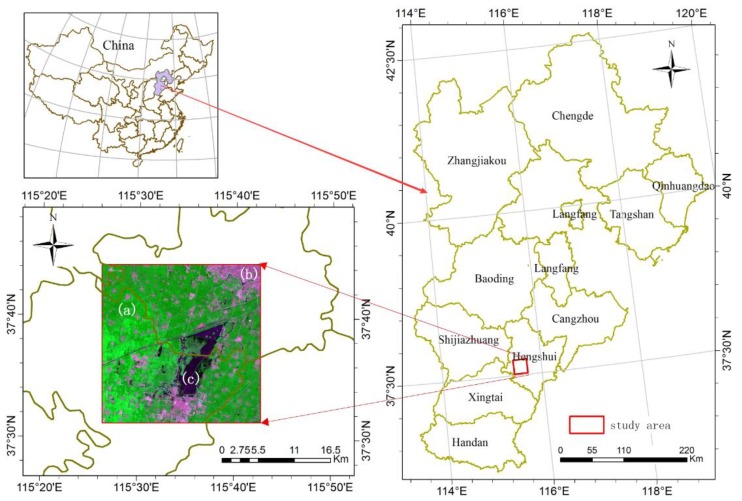
The location of study area.

The study region is located in the territory of Hengshui, Hebei Province in China (37.52°–37.73° N, 115.44°–115.71° E) (Figure 2), and covers approximately 25 km × 25 km. This region is dominated by fragmented cultivated land as shown in Figure 2a that is mixed with small areas of urbanized land (Figure 2b) and water bodies (Figure 2c). Broken parcels of cultivated land in this area are a typical feature because small land parcels are the basic management unit for farmers in China’s agricultural policy. These characteristics make this area a good place to test the applicability of USTARFM to identify fragmented agricultural units in the landscape.

The study dataset includes two Landsat 8 OLI images and two date 500 m daily MODIS surface reflectance products (MOD09GA) acquired on 19 August and 4 September 2014 (Table 1), which were downloaded from the USGS website (URL = http://earthexplorer.usgs.gov/).

**Table 1:** The main characteristics of Landsat 8 and MODIS data.

Data	Acquisition Date	(Path/Row)	Data Usage
Landsat 8 (OLI)	8/19/2014	123/034	Classification and similar pixels selection ($t_{k}$)
	9/4/2014		Accuracy assessment ($t_{0}$)
MOD09GA	8/19/2014	h27/v05	Unmixing data acquisition
	9/4/2014		

Images from these dates are of good quality and represent clear sky conditions. The Landsat 8 data products have been processed in geometric correction based on the terrain data. The main pre-processing included two steps: radiance calibration and atmospheric correction, which were conducted using FLAASH tools [36], then capable of comparing with the corresponding band of MODIS [26]. MOD09GA is the standard surface reflectance and provides MODIS band 1-7 daily surface reflectance at 500 m resolution accompanied with high-precision geolocation (approximately 50 m at NADIR) [1]. These MODIS images were re-projected from the native SIN projection to a UTM-WGS84 geospatial system and sampled by means of the MRT (MODIS Re-projection Tool). Similar to previous research, no further geometric correction was needed because the MODIS data were assumed to be well co-registered and matched the geographical position with the Landsat 8 data [1]. Additionally, compared to the models of bilinear filtering and cubic convolution, nearest neighbor resampling is an effective method to inherit the maximum original remote sensing spectral value. The MODIS at 500 m resolution were resampled into 480 m in order to match 16 times of 30 m of Landsat images, which facilitated the subsequent decomposition of MOD09GA pixels [1,31]. The green, red, and near-infrared bands, like [1,30], were chosen to test USTARFM performance.

### 3.2. Implementation Procedure

The ISODATA algorithm was applied to cluster a Landsat image collected August 19, generating land cover component maps with class number (*k*) as [5,10,15,20,25,30]. Two kinds of window scales which have pronounced impacts on the performance of the USTARFM method are concerned: the optimized window (*W*, MODIS pixel) for unmixing MODIS spectrum and the window (*w*, Landsat pixel) of searching the similar pixels for STARFM and USTARFM. For the *W*, a series of window size scales, [5 × 5, 7 × 7, 11 × 11, 15 × 15, 21 × 21, 31 × 31, 41 × 41], are used to unmix the MODIS data acquired on Aug. 19 at different class numbers, respectively. Next, the correlation coefficient (*γ*), RMSE and ERGAS were iteratively tested to unmix MODIS images to achieve the best results. This was completed as the popular benchmark to measure the difference between the reference Landsat 8 image and the unmixing data at different window sizes (*W*) and class number (*k*), and the optimal combination of the two parameters. An assumption considered here was that the relationship between land surface and landscape derived from the Landsat data are stable under the condition of the same area and same sensor [31]. So, the optimal window size determined from the 19 August 2014 images was inherited and further used to unmix the MODIS data acquired on 4 September 2014. The unmixing data for 19 August 19 and 4 September 2014 and the base date Landsat 8 data for 19 August 2014 were used together to predict the Landsat-like images on 4 September 2014 by weighting the similar pixels within a certain window size. The window sizes *w* (Landsat OLI pixel) were set as [7 × 7, 11 × 11, 31 × 31, 61 × 61, 101 × 101, 151 × 151].

The performance of the synthetic images were assessed using the correlation coefficient (*γ*) and *p*-value, RMSE, and ERGAS metrics [20,22]. The *γ* indicates the linear correlativity between fusion data and the reference data, and higher *γ* values represent better correlation between synthetic images and reference images; lower RMSE values show the lower fusion errors of synthetic images. In addition, ERGAS is a common comprehensive evaluation index for assessing the quality of a synthetic image which is able to quantity the spatial similarity degree of the fusion image and reference image [18,37]. These assessment metrics have different focus to show the performance of the synthetic images as shown in Equations (4)–(6):

(4) $$\gamma =\frac{\sum_{j=1}^{N}\left(x_{j}-\bar{x}\right)\left(y_{j}-\bar{y}\right)}{\sqrt{\sum_{j=1}^{N}{\left(x_{j}-\bar{x}\right)}^{2}.\sum_{j=1}^{N}{\left(y_{j}-\bar{y}\right)}^{2}}}$$

(5) $$\text{RMSE}=\sqrt{\frac{\sum_{j=1}^{N}{\left(x_{j}-y_{j}\right)}^{2}}{N}}$$

where *N* refers to the pixels number of fine-resolution image; *x_j_* and *y_j_* refer to the *j_th_* pixel values of the synthetic and reference images, respectively; and $\bar{x}$ and $\bar{y}$ are the mean value of synthetic data and reference data, respectively. ERGAS is calculated as:

(6) $$\text{ERGAS}=100\frac{H}{L}\sqrt{\frac{1}{N_{ban}}\sum_{i=1}^{N_{ban}}{\left(RMSE_{i}/M_{i}\right)}^{2}}$$

where *H* is the fine-resolution pixel size, such as 30 m; *L* is the coarse-resolution pixel size (480 m); *N_ban_* refers to the band number; *RMSE* refers to the *i_th_* band between the synthetic and reference data; and *M_i_* refers to the mean spectrum value of reference data in *i_th_* band.

## 4. Results and Discussion

### 4.1. Algorithm Performance Analysis Influenced by W, k and w

We tested whether USTARFM is an improved STARFM algorithm and compared the results with STARFM to determine whether improvements could be obtained from this algorithm.

**Table 2:** The accuracy of MODIS unmixing data on 19 August and 4 September 2014 at different combination of window size scales *W* and class number *k*.

Date	*W*	*k*	*γ*			RMSE			ERGAS		
			Green	Red	NIR	Green	Red	NIR	Green	Red	NIR
8/19/2014	7	5	**0.69**	**0.74**	**0.92**	**0.0204**	**0.0233**	**0.0378**	**2.0467**	**2.6494**	**0.7457**
		10	0.64	0.70	0.86	0.0218	0.0254	0.0511	2.1818	2.8778	1.0087
		15	0.49	0.55	0.76	0.0280	0.0339	0.0719	2.7995	3.8519	1.4185
		20	0.49	0.55	0.70	0.0274	0.0343	0.0850	2.7452	3.8968	1.6775
		25	0.33	0.41	0.44	0.0393	0.0469	0.1682	3.9298	5.3230	3.3205
		30	0.27	0.36	0.37	0.0455	0.0534	0.2117	4.5525	6.0575	4.1790
	11	5	0.75	0.80	**0.94**	0.0192	0.0212	**0.0334**	1.9256	2.4074	**0.6586**
		10	**0.78**	**0.82**	0.90	**0.0186**	**0.0205**	0.0436	**1.8594**	**2.3230**	0.8598
		15	0.66	0.70	0.88	0.0216	0.0255	0.0480	2.1587	2.8978	0.9484
		20	0.57	0.61	0.74	0.0244	0.0301	0.0779	2.4440	3.4197	1.5382
		25	0.49	0.55	0.47	0.0283	0.0345	0.1586	2.8297	3.9100	3.1305
		30	0.44	0.50	0.41	0.0308	0.0382	0.1950	3.0807	4.3364	3.8494
	15	5	0.76	0.81	**0.95**	0.0191	0.0210	**0.0321**	1.9126	2.3859	**0.6342**
		10	**0.82**	**0.86**	0.92	**0.0179**	**0.0189**	0.0399	**1.7908**	**2.1473**	0.7884
		15	0.74	0.78	0.92	0.0195	0.0218	0.0395	1.9489	2.4708	0.7790
		20	0.68	0.73	0.75	0.0208	0.0242	0.0755	2.0831	2.7472	1.4906
		25	0.61	0.67	0.47	0.0230	0.0274	0.1596	2.3025	3.1142	3.1503
		30	0.56	0.61	0.40	0.0250	0.0309	0.1980	2.5007	3.5034	3.9086
	21	5	0.77	0.81	**0.95**	0.0190	0.0210	**0.0313**	1.9047	2.3805	**0.6171**
		10	**0.85**	**0.88**	0.92	**0.0174**	**0.0179**	0.0387	**1.7426**	**2.0301**	0.7636
		15	0.80	0.85	0.94	0.0181	0.0189	0.0338	1.8075	2.1443	0.6671
		20	0.76	0.81	0.75	0.0189	0.0209	0.0762	1.8915	2.3697	1.5033
		25	0.72	0.77	0.47	0.0199	0.0226	0.1734	1.9972	2.5679	3.4237
		30	0.68	0.71	0.39	0.0211	0.0251	0.2074	2.1118	2.8530	4.0942
	31	5	0.77	0.81	**0.95**	0.0190	0.0212	**0.0297**	1.9021	2.3749	**0.5360**
		10	**0.86**	**0.90**	0.92	**0.0171**	**0.0173**	0.0391	**1.7107**	**1.9402**	0.7714
		15	0.81	0.87	0.95	0.0178	0.0180	0.0305	1.7796	2.0228	0.6023
		20	0.83	0.88	0.70	0.0173	0.0177	0.0877	1.7368	1.9883	1.7310
		25	0.78	0.84	0.43	0.0185	0.0194	0.1857	1.8486	2.1757	3.6662
		30	0.78	0.82	0.37	0.0185	0.0202	0.2254	1.8540	2.2664	4.4490
	41	5	0.75	0.80	**0.95**	0.0193	0.0214	**0.0300**	1.9360	2.4314	**0.5446**
		10	**0.85**	**0.89**	0.92	**0.0173**	**0.0174**	0.0387	**1.7310**	**1.9755**	0.7643
		15	0.81	0.88	0.95	0.0178	0.0178	0.0401	1.7852	2.0206	0.5926
		20	0.85	0.89	0.70	0.0173	0.0174	0.0911	1.7965	1.9787	1.7981
		25	0.80	0.87	0.43	0.0180	0.0184	0.1956	1.8047	2.0834	3.8616
		30	0.82	0.86	0.34	0.0176	0.0186	0.2704	1.7598	2.1079	5.3374
9/4/2014	31	10	0.82	0.86	0.90	0.0182	0.0222	0.0401	1.8052	2.3020	0.8737

Note: Underlined bold values indicate the best value to determine the optimal window size (*p*-value < 0.001).

The unmixed image as traditional Unmixing model is also produced during the USTARFM process, so we put the three fusion methods together to achieve a more comprehensive understanding of the synthetic image performance. Table 2 shows the performances of the three fused methods with varying *W* and *k* settings using the metrics of *γ*, RMSE and ERGAS as benchmarks to determine the optimal combination of window size and class number for unmixing MODIS images (the best options are underlined and bold in Table 2). For the green and red bands, the optimal combination of window size (*W*) and class number is 31 × 31 and 10, and for NIR, the window size (*W*) and class number are 31 × 31 and 5, respectively. Considering that the metrics for the NIR band are similar to the green and red band, and more classes are feasible to guarantee class features uniform, we chose the window size of 31 × 31 MODIS pixels and the class number of 10 as the optimal combination for further unmixing MODIS data acquired Sept. 4.

From the above analysis, the *W* and *k* were determined. The next step was to focus on how to calculate *w* for USTARFM. Table 3 shows the performance of the USTARFM and STARFM methods at different window sizes (*w*) to search the similar pixels for the central pixel. The USTARFM algorithm achieves the optimal effect when the window size *w* is 11 × 11 OLI pixels for red and green bands, and 31 × 31 OLI pixels for the NIR band. Compared to 31 × 31 for the STARFM, the difference of optimal window size between NIR and visible bands may be caused by their reflectance characteristics which affect the similar pixels search efficiency. Relative to NIR, the green and red are the short-wave bands, which are sensitive to weather related factors, such as haze.

Though the atmosphere process has been applied for MODIS data, the atmosphere effects cannot be entirely removed [38]. For the other two bands, the indices for the three methods have the same trend as the green band. Table 3 also illustrates that the best window size of USTARFM is smaller than that of STARFM, and at the same window size, the three indices of USTARFM are also better than those of STARFM. This is mainly because the unmixing data used in USTARFM is efficient to reflect the surface reflectance comparing to the directed resampled data used in STARFM.

**Table 3:** The accuracy of the STARFM and USTARFM at different window size scales.

Method	Window Size *w* n × n OLI Pixels	*γ*			RMSE			ERGAS		
		Green	Red	NIR	Green	Red	NIR	Green	Red	NIR
STARFM	7	0.8822	0.8926	0.9416	0.0130	0.0172	0.0373	1.2823	1.8061	0.8088
	11	0.8880	0.8987	0.9394	0.0130	0.0172	0.0351	1.2865	1.8129	0.7611
	31	**0.8895**	**0.9000**	0.9489	**0.0127**	**0.0170**	**0.0300**	**1.2559**	**1.7916**	**0.6507**
	61	0.8844	0.8948	**0.9490**	0.0131	0.0176	0.0302	1.2968	1.8498	0.6553
	101	0.8804	0.8931	0.9474	0.0133	0.0177	0.0309	1.3117	1.8673	0.6702
	151	0.8792	0.8921	0.9475	0.0133	0.0179	0.0310	1.3147	1.8782	0.6735
USTARFM	7	0.9116	0.9226	0.9600	0.0118	0.0151	0.0260	1.1678	1.5876	0.5654
	11	**0.9129**	**0.9229**	0.9631	**0.0116**	**0.0151**	0.0249	**1.1502**	**1.5850**	0.5416
	31	0.9121	0.9192	**0.9650**	0.0117	0.0154	**0.0245**	1.1550	1.6224	**0.5317**
	61	0.9106	0.9171	0.9650	0.0117	0.0156	0.0245	1.1564	1.6437	0.5326
	101	0.9094	0.9158	0.9650	0.0117	0.0158	0.0246	1.1615	1.6572	0.5334
	151	0.9083	0.9145	0.9650	0.0118	0.0159	0.0246	1.1671	1.6700	0.5341

Note: Underlined bold values indicate the best value to determine the optimal window size (*p*-value < 0.001).

### 4.2. Accuracy Assessment Under the Best Parameters Setting

For the green band at the optimal window size (*W* = 31 × 31), the *γ* = 0.91 of the USTARFM method is higher than those of the STARFM (0.89) and the unmixing method (0.82); meanwhile, USTARFM shows the lowest error (RMSE = 0.0116) compared to the prediction of the STARFM (RMSE = 0.0127) and the unmixing method (RMSE = 0.0182); ERGAS shows the same trend (1.1502 *vs.* 1.2559 *vs.* 1.8052, respectively).

**Figure 3:**
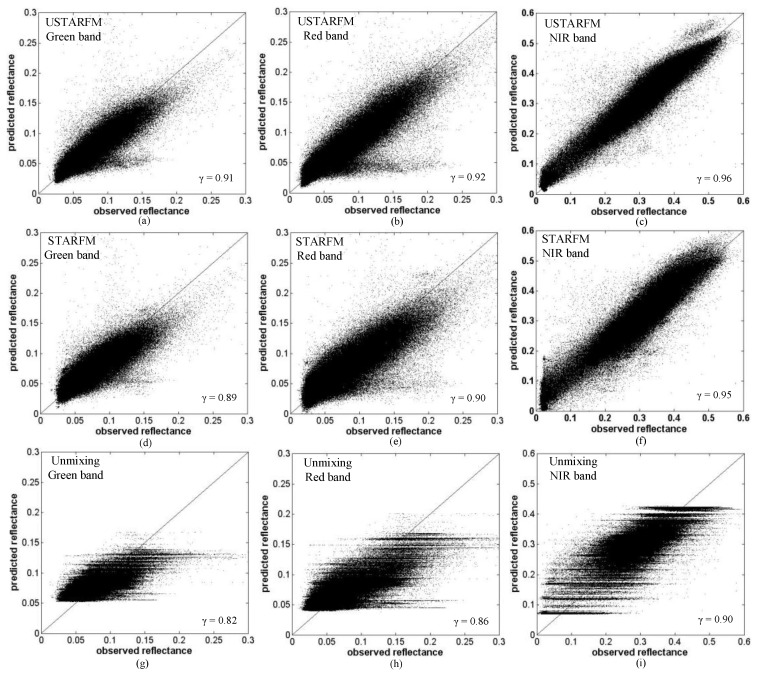
Scatterplots of the real reflectance and the predicted product produced by the three algorithms for the green, red and NIR-infrared bands.

Figure 3 shows the scatterplots between reflectance estimate from USTARFM, STARFM and unmixing based method, with the optimized parameter setting, (*W*, *k*) and *w*, and the corresponding reference bands of Landsat 8 image. USTARFM inspired from STARFM improves the performance of central pixel reflectance estimation by weighting the neighboring similar pixels within the moving window, which reflected the spatial variability well, producing scatter point distributions close to 1:1. The *γ* of each band of images generated by USTARFM and STARFM is superior to that of the unmixing method, by about 0.02. The reason is that the reflectance calculated by the unmixing method is the mean reflectance of each clustered class within the window, which blurs the spatial heterogeneity and makes the scatterplot appear in “stripe” pattern (Figure 3g–i). Generally, the images at Landsat-like image predicted by the USTARFM are more accurate than those derived from the STARFM and the unmixing methods.

### 4.3. Landscape Heterogeneity Impact on USTARFM Performance

The heterogeneity over the regions where complex mixtures of land-cover type commonly exist represents a big challenge for STAFRM to identify pure pixels [25,30]. To analyze the performance of the USTARFM algorithm in a complex landscape, the MODIS pixel scale, 480 m × 480 m, was defined as a grid to divide the study area, and each grid was marked according to its heterogeneous degree. Here, we developed the degree of heterogeneity (*h*) metric using the clustered of thematic map from the Landsat 8 image as described in Equation (7). This idea is based on the quantity of land cover classes in one grid. More than one class type in one grid implies a higher degree of heterogeneity . If (*N_c_* > 0) (*N_c_* is the pixel number of class *c*) is a determinant function to judge whether class *c* appears in the grid. *P* is marked as 1 if *N_c_* is more than 1:

(7) $$h=\sum_{c=1}^{Num}\left(P,if(N_{c}>0)\right)$$

**Figure 4:**
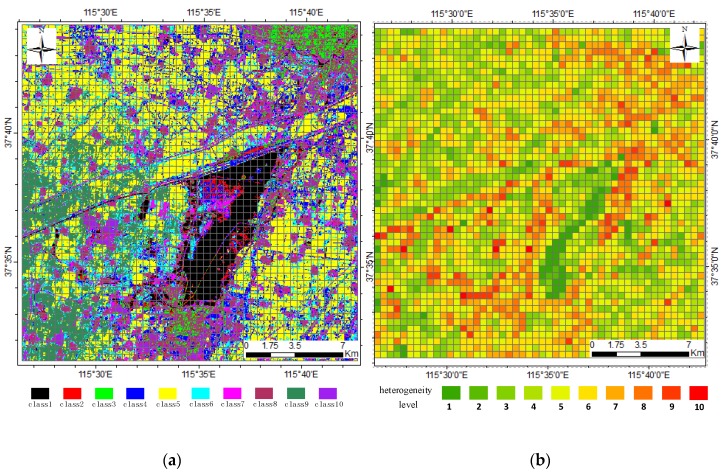
The distribution of different heterogeneity levels in the study area. (**a**) class types within a grid (MODIS pixel scale), (**b**) the heterogeneity levels of MODIS pixels.

*Num* is the total class in the thematic map, here maximum number of the land cover type is 10. From this definition of the heterogeneity metric, the heterogeneity level of a grid with only one class is defined as 1 which represents the lowest heterogeneous degree. Otherwise, the higher the number of classes that appear in a pixel, the higher the degree of heterogeneity. The highest level of heterogeneity assigned was 10 (Figure 4). From Figure 2 and Figure 4, it can be seen clearly that the areas of higher heterogeneity are distributed from the north east to south west area where fragmented cultivated land is common. The areas of lower heterogeneous grids were concentrated in the northwest which is dominated by larger cultivated fields.

**Figure 5:**
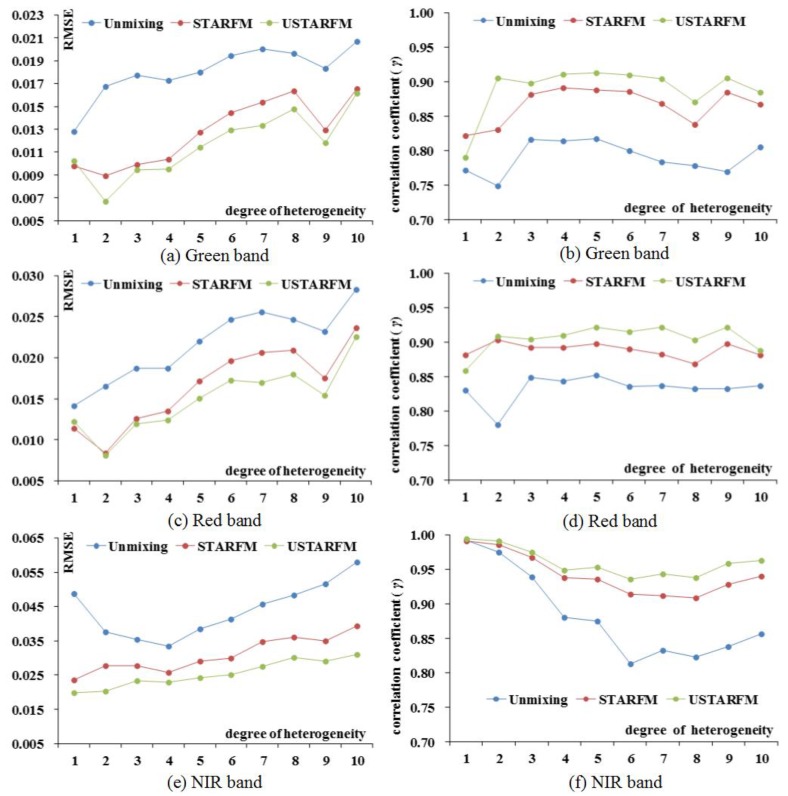
*γ* and RMSE of the three methods at different heterogeneity levels.

Figure 5 shows the RMSE and *γ* of Green, Red and NIR data generated by the three fused methods at different heterogeneity levels. For the three bands, the change trend decreases is accompanied by the heterogeneity degree increases, highlighting that the heterogeneity is a sensitive variable for the STARFM and has side-effects on the performance of the proposed method. However, an interesting result is that higher performance was achieved from USTARFM than those of STARFM at each heterogeneity level, except for level one (Figure 5a–d).

Table 4 analyzes the relationship of the unmixing and resampled MODIS data at different heterogeneity levels to the Landsat reference data on 4 September 2014, which illustrates why USTARFM is superior to the STARFM. The relationship was obviously higher from 2 to 10 level, consistent with the trend of USTARFM trend. This is because the unmixing data instead of directed sampling data as basis for USTARFM can describe the mixed landscape clearly, especially in the higher heterogeneity level area, while the resampled method just offer the STARM uniform spot without reflecting the land cover heterogeneous characteristics. However, the areas at the heterogeneity level of 1 are special as the most homogeneous region, where STARFM has better performance. The USTARFM precedes the unmixing method but is inferior to STARFM for the green and red bands. Taking the result of the green band as an example, the RMSE and *γ* of the green band predicted by the STARFM are better than those for USTARFM (for green, RMSE: 0.0097 *vs.* 0.0102; *γ*: 0.82 *vs.* 0.79). This is because the accuracy of the directly resampled data in the homogeneous area for the STARFM is better than the unmixing data for USTARFM (for green, *γ*: 0.89 *vs.* 0.77; RMSE: 0.0102 *vs.* 0.0128) (Table 4), resulting in the lower accuracy of the proposed method.

**Table 4:** The relationship of data generated from the unmixing method and resampled data to the reference data at different heterogeneity levels (*h*).

	*h*	Unmixing Data			Resampled Data		
		Green	Red	NIR	Green	Red	NIR
γ	1	0.77	0.83	0.99	0.89	0.90	0.98
	2	0.75	0.80	0.98	0.72	0.75	0.96
	3	0.82	0.85	0.94	0.74	0.76	0.91
	4	0.81	0.84	0.88	0.67	0.69	0.80
	5	0.82	0.85	0.87	0.52	0.53	0.73
	6	0.80	0.84	0.81	0.43	0.42	0.58
	7	0.78	0.84	0.83	0.33	0.33	0.51
	8	0.78	0.83	0.82	0.26	0.25	0.36
	9	0.77	0.83	0.84	0.17	0.20	0.19
	10	0.81	0.84	0.86	0.11	0.11	0.11
RMSE	1	0.0128	0.0142	0.0487	0.0102	0.0111	0.0492
	2	0.0167	0.0165	0.0377	0.0158	0.0152	0.0448
	3	0.0177	0.0187	0.0355	0.0183	0.0200	0.0433
	4	0.0173	0.0187	0.0334	0.0198	0.0231	0.0412
	5	0.0180	0.0220	0.0384	0.0243	0.0327	0.0530
	6	0.0194	0.0247	0.0412	0.0271	0.0380	0.0574
	7	0.0200	0.0256	0.0456	0.0287	0.0409	0.0711
	8	0.0196	0.0247	0.0484	0.0287	0.0407	0.0803
	9	0.0184	0.0232	0.0517	0.0270	0.0387	0.0969
	10	0.0207	0.0283	0.0580	0.0343	0.0505	0.1187
ERGAS	1	1.4521	1.9675	1.7721	1.1564	1.5454	2.3189
	2	2.1569	2.6678	0.8700	2.0340	2.4532	1.0356
	3	2.1529	2.7151	0.7056	2.2202	2.9025	0.8611
	4	1.9872	2.4681	0.6794	2.2763	3.0501	0.8381
	5	1.7909	2.3277	0.8090	2.4146	3.4571	1.1177
	6	1.7458	2.2490	0.8931	2.4326	3.4586	1.2431
	7	1.7065	2.1696	1.0584	2.4477	3.4721	1.6493
	8	1.6423	2.0622	1.1664	2.3994	3.4034	1.9355
	9	1.6074	2.0815	1.2301	2.3673	3.4731	2.3083
	10	1.6290	2.1737	1.4136	2.6959	3.8751	2.8950

**Figure 6:**
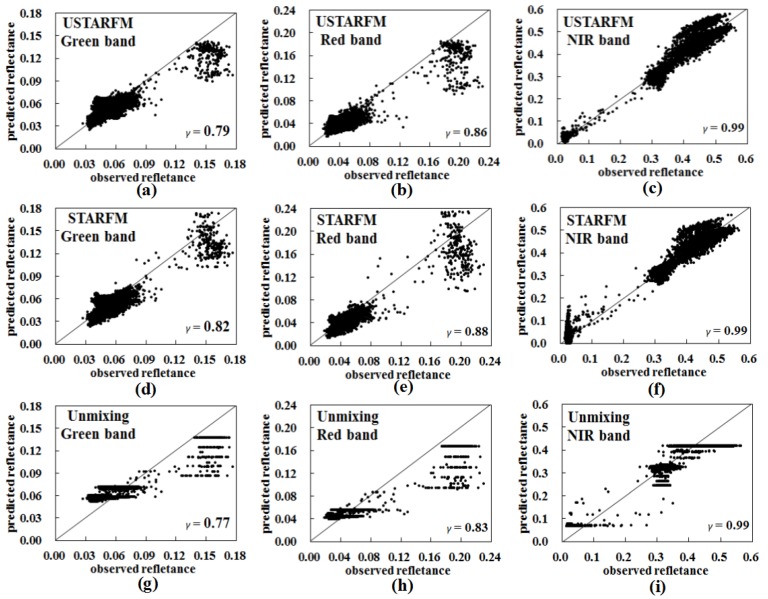
Scatterplots of three bands generated by the three methods in the region with the heterogeneity level of 1.

In Figure 6g, the higher reflectance values for red and green bands seem to be underestimated in some scatter points (residential region, a MODIS pixel scale), which may be because the pure coarse pixel was affected by the surrounding pixels when it is decomposed in Equation (1), and the error of the unmixing data causes accumulated error in the USTARFM (Figure 6a). However, for the NIR band, the accuracy of the unmixing data is slightly better than directly resampled data in the homogeneous area (*γ*: 0.99 *vs.* 0.98; RMSE: 0.0487 *vs.* 0.0492), which makes the RMSE of the USTARFM lowest compared to the STARFM and unmixing methods (0.0198 *vs.* 0.0235 *vs.* 0.0487) (Figure 5e) and the scatterplot more close to 1:1 (Figure 6c). This is mainly because, relative to the green and red, NIR is the long-wave bands, which are not sensitive to weather related factors such as haze and have a high signal-to-noise when they are decomposed linearly [37].

### 4.4. Synthetic Image Analysis

**Figure 7:**
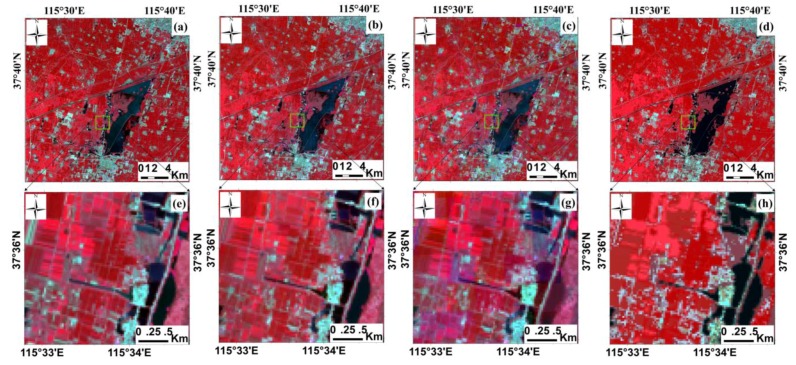
The comparison of real and fusion images produced by the three algorithms (NIR-red-green combination). (**a**) shows the reference Landsat 8 images observed on 4 September 2014; (**b**–**d**) are the prediction images by USTARFM, STARFM, and unmixing-based method, respectively; (**e**–**h**) indicate the enlarged subset images of (**a**–**d**), respectively.

The synthetic images of these three methods look similar to the reference Landsat 8 images acquired 4 September 2014 (Figure 7a). Figure 7b–d shows that land cover with large area, e.g., water bodies, residential areas and wide roads, can be clearly identified. From the subplots of the synthetic images (Figure 7f–h), the synthetic image in the heterogeneous region produced by the unmixing method appears somewhat “blurry” and a homogeneous “plot” in which we could not discern the small objects. In contrast, areas from the images generated by the USTARFM and STARFM represent more spatial details (Figure 7g–h). Compared with the image predicted by STARFM (Figure 7g), the result produced by the USTARFM has a lower spectral deviation to the actual spectrum (Figure 7e) in the fragmentation region. This is because the directly resampled data used in STARFM has uniform spectral reflectance, which is unable to reveal the heterogeneity within the MODIS pixel.

### 4.5. Algorithm Applicability Analysis

We analyzed another series of study dates to test the applicability of USTARFM. This study area is situated in a region of Virginia, in the Eastern United States (about 37.51°–37.72° N and 76.99°–77.26° W) where there are many small patches of different land types including forest, bare soil, and residential patches. The dataset includes Landsat 7 ETM+ and MOD09GA images acquired for 25 January, 26 February, and 17 May, in 2002. In the study, the Landsat 7 ETM+ and MOD09GA for 25 January is the base data for predicting the Landsat-like data collected 26 February and 17 May 2002. The dataset has been applied for data fused study, and the detailed description about study area and dataset can be found in publication [30].

The series parameters for the USTARFM were the same as for the previous experiment. Through analyzing the unmixed results of MODIS data for data acquired 25 January 2002, the optimal combination of window size and the class number (*W* = 31, *k* = 10) were also determined for unmixing the MODIS data acquired for 26 February, and 17 May 2002. Then these unmixed data are input to USTARFM for predicting the surface reflectance for data collected 26 February and 17 May 2002. The result for 26 February 2002 shows the accuracy of USTARFM is better than STARFM (*i.e.*, for green band, the *γ*: 0.92 *vs.* 0.90; RMSE: 0.0097 *vs.* 0.0116; ERGAS: 0.9058 *vs.* 1.0797), which achieved higher accuracy at a smaller window size compared to STARFM (*w* = 15 × 15 *vs.* 45 × 45). The predicted reflectance of 17 May 2002 by USTARFM are nearly the same for the unmixing method, and are still better than STARFM (*i.e.*, for green band, the *γ*: 0.80 *vs.* 0.83 *vs.* 0.74; RMSE: 0.0159 *vs.* 0.0148 *vs.* 0.0178; ERGAS: 1.4529 *vs.* 1.3487 *vs.* 1.6289). However, the performance of the predicted result is lower than those acquired on 26 February 2002, because some changes of land-cover types from the larger time span occurred, which is also consistent with Zhu’s result [30].

**Figure 8:**
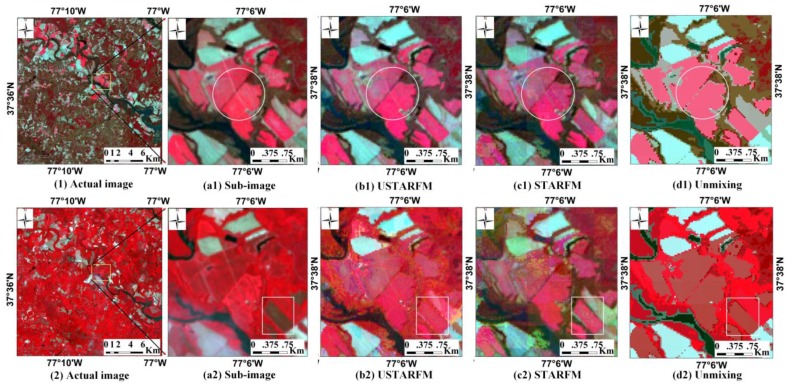
Landsat images collected 26 February 2002 (1) and May 17 (2) and their enlarged sub-images (**a1**) and (**a2**); and the corresponding prediction sub-images by USTARFM (**b1,** **b2**), STARFM (**c1,** **c2**), and unmixing-based method (**d1,** **d2**).

Figure 8 shows the synthetic images of 26 February and 17 May 2002 for the three methods. The prediction image by USTARFM (Figure 8(b1)) on 26 February 2002 is almost the same as that by STARFM (Figure 8(c1)) visually, but the quantitative evaluation of the former is better than the latter, and the unmixed image is worse for loss of texture details of surface ground in Figure 8(d1) (marked in circle). However, due to land cover changed over the long time span that single date Landsat was unable to record the changes, it was also unfulfillable to reflect the changed information from the fusion data [25]. This is why, in this paper, the predicted images from 17 May 2002 by USTARFM and STARFM have some variances from the actual image shown in Figure 8(a2) (marked by a rectangle). Therefore, the STAARCH model, proposed by Hilker [28], will offer us some new ideas to solve the problem. Besides, the high temporal frequency MODIS at long time scale can record the land surface changed trace, which may be a good alternative to reflect the crop changes in agricultural landscape.

## 5. Conclusions

The temporal-spatial fusion of remote sensing data is an effective approach to solve the dilemma of the simultaneous attainment of high temporal and spatial resolution remote sensing images. In this study, an USTARFM algorithm based on improved STARFM supported by an unmixing-based method was developed to generate high-temporal-spatial remote sensing data. The USTARFM algorithm was tested by the experimental data from Landsat 8/Landsat 7 and MODIS images in two study areas.

USTARFM adopts the unmixed fraction from the unmixing data, which is able to reflect surface information in heterogeneous areas, to take place of the directed resampled data used for STARFM, which can increase the probability of searching the “pure pixels” and guarantee the fusion accuracy. This enables the USTARFM to achieve better performance than STARFM at the same window size, even smaller (*w*, 11 × 11 *vs.* 31 × 31). In the Hengshui study area, taking the predicted NIR band reflectance as an example, with the best combination of window size, class number and similar pixels searching window, *γ* of the USTARFM, STARFM and unmixing were 0.96, 0.95, 0.90, respectively (*p*-value < 0.001); RMSE were 0.0245, 0.0300, 0.0401, respectively; and ERGAS were 0.5416, 0.6507, 0.8737, respectively. However, the scatterplots of synthetic data produced by USTARFM are more close to 1:1. The synthetic images produced by the USTARFM look more similar to the reference images in RGB combination manner (Figure 7). Especially in the broken area, USTARFM is skilled in reducing the homogeneous “plot” generated by the unmixing method and maintains the fidelity of the spectrum. In addition, the similar conclusions for USTARFM were also obtained for a second study area in Virginia which is a relatively heterogeneous area.

The influence of heterogeneity on the three methods shows the certain regularity (Figure 5). The USTARM showed consistent higher performance than those of STARM, except for the Red and Green bands when heterogeneity degree as 1, highlighting that this method is capable of solving the problem of fused data that is present in STARM. It is an exciting result that, for the NIR band, the USTARFM is performs consistently better than STARFM when heterogeneity degree ranges from 1 to 10. Compared to the short-wave band as Red and Blue, the long-wave band, NIR, it is insensitive to the atmospheric effect to ensure the unmixing result, even in the area when heterogeneous degree is 1.

On the whole, USTARFM works better than STARFM and the unmixing based method. As the heterogeneity increased, the USTARFM was better than the STARFM and unmixing method. But if the land-cover patches are “extremely” broken and the high-spatial remote sensing images are unable to capture the texture details, it is still difficult to achieve an acceptable result.

There are some issues that need to be addressed in further study. First, the accuracy of the USTARFM depends on the performance of the unmixing data generated from Equation (1). The USTARFM method could get better results depending on the higher precision unmixing data. How to achieve better unmixed data from development of unmixing model or finer remote sensing is still an important research topic. Next, the land-cover types changed greatly over the long time span of this research, the performances of USTARFM and STARFM are degraded, such as 0.12 and 0.16 of correlation coefficient in green band lower than usual analyzed in Virginia area in this study. This problem urgently needs to be addressed because this affects the ability of USTARFM to detect cultivated land where usually had abrupt change at short temporal scale. Third, the co-registration errors between Landsat 8 and MODIS is still potential factor with side-effect on the fusion result, which are usually neglected. Previous researchers have done related research on the influence of co-registration errors on data fusion [39,40]. Maybe, this is another way needing to be concerned to improve USTARFM performance. Last but not least, the applicability of the USTARFM method in inversing parameters, such as e.g. NDVI or biomass, need to be further tested. Although the work in this paper is based on Landsat and MODIS surface reflectance data are popular satellite sources, the USTARFM algorithm still has a prospect for other high resolution images (e.g., Chinese GF-1 satellite data with 16-m resolution, French SPOT-5/6satellite data with 10/6 m resolution, *etc.*) and medium resolution images (e.g., the NPOESS VIIRS data U.S. operational polar satellite constellation which is the new generation satellite after MODIS).
